# CT-Guided Online Adaptive Radiotherapy Delivered via Personalized Ultrafractionated Stereotactic Adaptive Radiotherapy (PULSAR) for a Bulky Thoracic and Abdominal Mass in Oligometastatic Renal Cell Carcinoma

**DOI:** 10.7759/cureus.67502

**Published:** 2024-08-22

**Authors:** Ari A Kassardjian, Colton Ladbury, Borna Maraghechi, Chengyu Shi, Tyler Watkins, An Liu, Kevin Tsai, Terence Williams, Yun Rose Li, Savita Dandapani, Amanda Schwer, Percy Lee

**Affiliations:** 1 Radiation Oncology, City of Hope National Medical Center, Duarte, USA; 2 Radiation Oncology, City of Hope Orange County Lennar Foundation Cancer Center, Irvine, USA

**Keywords:** ethos, peripheral lung sbrt, sbrt (stereotactic body radiotherapy), renal cell carcinoma (rcc), adaptive radiation therapy

## Abstract

In the context of oligometastatic renal cell carcinoma (RCC), local treatment with stereotactic body radiotherapy (SBRT) may improve oncologic outcomes. However, the location and size can often pose a technical challenge in standard SBRT delivery, and the dose is potentially limited by nearby organs at risk (OARs). Online adaptive radiotherapy (oART) improves radiation delivery by personalizing high-dose fractions to account for daily stochastic variations in patient anatomy or setup. The oART process aims to maximize tumor control and enhances precision by tailoring to a more accurate representation of a patient in near-real time. The proceeding re-optimization can mitigate the uncertainty inherent in the traditional radiation delivery workflow and precludes the need for larger margins that account for anatomical variations and setup errors. Here, we describe a case of oligometastatic RCC with a bulky (>300 cm^3^) pleural-based left lower lobe mass extending into the upper abdomen treated via personalized ultrafractionated stereotactic adaptive radiotherapy (PULSAR). Three fractions were delivered four weeks apart allowing for tumor shrinkage of these bulky lesions, and oART permitted on-table adaptation of the plan without traditional re-simulation and re-planning required during off-line adaptive radiotherapy. The plan was designed for the Ethos linear accelerator (Varian Medical Systems, Inc., Palo Alto, CA, USA). The prescription dose was 36 Gray (Gy) in three fractions, and the adapted plan was selected in each treatment over the scheduled plan due to better target coverage and reversal of OAR dose violations. The adapted plan met all OAR dose constraints, and it achieved higher target coverage in the first two PULSAR fractions compared to the scheduled plan. In the third fraction, the cumulative point dose was approaching the maximum heart tolerance, and target coverage was accordingly compromised based on clinical judgment. There was evidence of tumor regression throughout the course of treatment, and the patient did not develop any significant radiation-related toxicities. Follow-up imaging has demonstrated the overall stable size of her lesion without any evidence of disease progression. Our case reflects the benefit of adaptive SBRT delivery to a bulky mass near multiple OARs in the setting of oligometastatic RCC. The adapted plan allowed for prioritization of critical structures on a fraction-by-fraction basis while preserving the therapeutic intent of SBRT. Further integration of advanced imaging techniques, optimal disease-specific systemic immunotherapies or targeted therapies, and refinement of patient selection will be crucial in identifying which patients would most benefit from an adaptive approach.

## Introduction

The treatment approach for advanced-stage renal cell carcinoma (RCC) historically has involved a combination of surgery and systemic therapy. Radiation therapy was often overlooked in part due to the perceived radioresistant nature of RCC [1]. With older techniques, dose escalation to overcome radioresistance often resulted in an unacceptable dose and toxicity to nearby organs at risk (OARs). Stereotactic body radiotherapy (SBRT) allows for conformal delivery of ablative doses to maximize the biologically effective dose (BED) while minimizing the dose to nearby critical organs at risk (OARs). More recently, SBRT was shown to have excellent local control (LC) and safety profile for both primary and oligometastatic RCC [2-4].

When considering SBRT for oligometastatic RCC, radiation oncologists face specific challenges based on sites of involvement. Given the high ablative dose characteristic of SBRT and its delivery over a small number of treatments, positional accuracy is crucial, and treatment planning must account for changes to both tumors and OARs. Online adaptive radiotherapy (oART) incorporates parallel processing and artificial intelligence to generate treatment plans using image-guided radiation therapy (IGRT) while a patient is on the treatment couch and hence more closely reflects anatomical shifts prior to delivery of a treatment fraction [5]. This combination of oART and SBRT allows for delivery of ablative doses with a higher degree of certainty, which potentially mitigates the dose to OARs.

Personalized, ultrafractionated stereotactic adaptive radiotherapy (PULSAR) represents a growing trend that employs longer times between fractions to enhance tumoral regression and sublethal repair of healthy tissues [6,7]. Rather than the standard practice of separating SBRT fractions by 24 or 48 hours, PULSAR can space SBRT fractions by weeks to months to stimulate immune responses and synergize with systemic immunotherapeutic agents [8,9]. This approach, given the fewer fractions and allowance for normal tissue recovery, may also be less immunosuppressive than conventionally fractionated radiotherapy. Here, we describe an oART plan of a bulky thoracic and upper abdominal mass in a 79-year-old patient with oligometastatic RCC delivered via PULSAR. She was successfully treated with three monthly fractions of SBRT to a large left-sided pleural-based lesion extending into the upper abdomen. Although early in follow-up, re-staging imaging has demonstrated stable disease, and the treatment was delivered with favorable tolerability and safety profile.

## Case presentation

**Figure 1:**
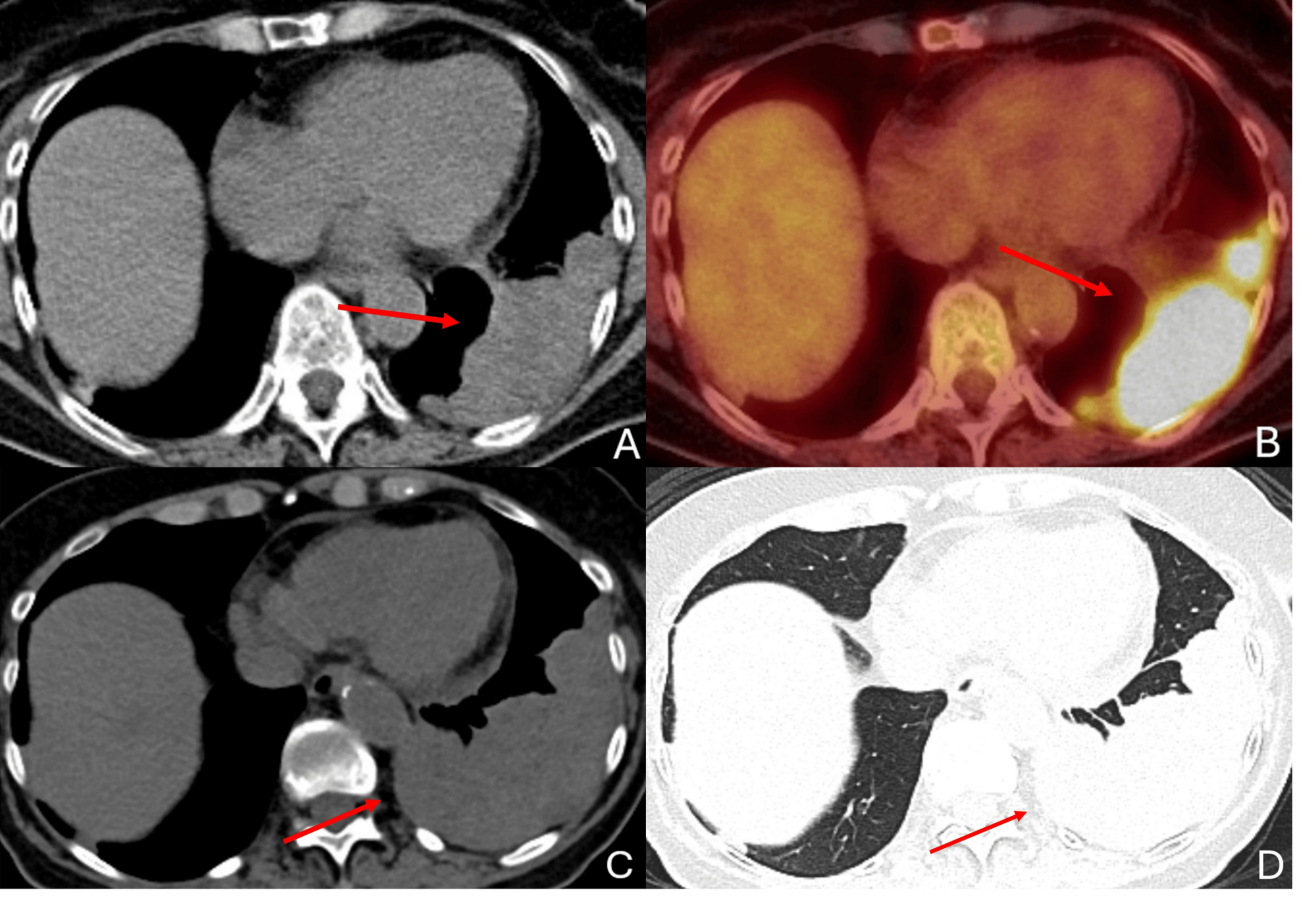
Diagnostic Computed Tomography Imaging of a Bulky Pleural-Based Mass Positron emission tomography-computed tomography (PET/CT) scan three months prior to treatment showing the transverse dimension on CT (A) with PET overlay (B) of the pleural-based mass extending into the upper abdomen. The mass measures 8.5 x 4.4 centimeters (cm) in the transverse dimension with a maximum standardized uptake value (SUV) of 16.5. There was evidence of progression seen on the non-contrast CT scan one week prior to treatment, measuring 11.4 x 5.3 cm in the transverse dimension and seen on mediastinal (C) and lung (D) windows. Red arrows indicate the left lower lobe (LLL) mass.

The patient initially underwent left-sided nephrectomy at age 61 for early-stage RCC. She was monitored for 17 years until she developed fatigue, dyspnea, and cough. Computed tomography (CT) of the chest showed a pleural-based mass in the left lower lung, measuring 5.8 x 3.5 centimeters (cm) in the transverse dimension. She was seen by pulmonary medicine and underwent robotic-assisted bronchoscopy of the left lower lobe (LLL). Surgical pathology was consistent with metastatic clear-cell RCC. Subsequent positron emission tomography-computed tomography (PET/CT) imaging revealed an interval increase in size of the LLL mass to 8.5 x 4.4 cm with a maximum standardized uptake value (SUV) of 16.5 (Figures 1A, 1B). The lesion extended through the diaphragm, and there was contiguous involvement with upper abdominal soft tissue. She initiated systemic treatment with ipilimumab and nivolumab and was planned to undergo SBRT to her bulky site of disease in the LLL for local control via PULSAR in three fractions delivered every four weeks. Prior to her treatment, she underwent diagnostic re-staging imaging, which demonstrated an interval increase in the size of her LLL pleural-based mass, now measuring 11.4 x 5.3 cm in the transverse dimension (Figures 1C, 1D).

A CT simulation scan was performed while the patient was supine using both free-breathing and deep inspiration breath-hold (DIBH) techniques. The initial reference plan was created based on the original planning CT scan (pCT) with contours created off-line by the treating radiation oncologist. When generating the reference plan from the pCT using a standard, non-adaptive approach, there were significant concerns given the large size of the bulky mass and its susceptibility to motion. With daily IGRT, a standard planning target volume (PTV) margin of 0.5 to 1.0 cm is often necessary to offset inter-fractional and setup uncertainties in the non-adaptive setting. Furthermore, delivery of ablative doses of SBRT would be constrained by the dose to nearby OARs, including great vessels, colon, spinal cord, esophagus, stomach, and heart.

In total, there were 323 cubic centimeters (cc) of the LLL lung and abdomen mass contoured as gross tumor volume (GTV). A 0.3 cm margin was applied to generate the PTV on the pCT for the reference plan. In total, the resulting PTV measured 477 cc and extended 20 cm in the craniocaudal direction. Optimization structures, GTVOpt and PTVOpt, were generated and used during the treatment planning process. GTVOpt was created by applying an internal 0.3 cm margin from the existing GTV. PTVOpt was generated by cropping the PTV 0.3 cm from the heart border. A provisional three-fraction plan was designed for the Ethos linear accelerator (Varian Medical Systems, Inc., Palo Alto, CA, USA) using adaptive SBRT. Ethos incorporates an oART workflow based on high-quality iterative cone-beam CT (CBCT) images and planning software [10]. Prior to delivery of each SBRT fraction, a CBCT is obtained, and a non-adapted, or scheduled plan is generated based on the unmodified reference plan applied to the CBCT. In contrast, the adaptive plan is calculated and re-optimized from the new contours drawn by the radiation oncologist based on the patient’s anatomy of the day.

The prescription dose was to deliver 36 Gray (Gy) in three fractions with over 95% of the PTV receiving at least 95% of prescription dose. Similar to the reference plan, the scheduled and the adapted plans used a 0.3 cm margin expanded from the CTV to create the PTV. The DIBH technique was used during oART to minimize the impact of respiratory motion on the pleural-based mass. Surface guidance was not available for use to monitor movement during treatment. The adapted plan was selected in favor of the scheduled plan in all three fractions given its superior PTV coverage and decrease in dose to OARs. In the first fraction of PULSAR, the dose to 95% of the PTV was 100.2% in the adapted plan, compared to 95.1% in the non-adapted scheduled plan (Table 1).

**Table 1:** Target Coverage and Dose Evaluation in Adapted and Non-adapted Plans Target coverage objectives and organ at risk (OAR) constraints with associated metrics for the reference plan, scheduled plan, and adapted plan. Target structures are gross target volume (GTV) and planning target volume (PTV). Coverage for optimization planning structures PTVOpt and GTVOpt are also included. Metrics are reported per fraction in addition to mean and standard deviation (SD) for scheduled and adapted plans.

			Scheduled Plan				Adapted Plan			
Structure	Objective	Reference Plan	Fraction 1	Fraction 2	Fraction 3	Mean ± SD	Fraction 1	Fraction 2	Fraction 3	Mean ± SD
GTV V100% (%)	≥ 95%	98.8	97.5	98.5	94.5	96.8 ± 2.1	99.6	99.6	49.7	83 ± 28.8
GTV V95% (%)	≥ 95%	99.5	99.4	99.7	97.3	98.8 ± 1.3	99.9	99.9	69.6	89.8 ± 17.5
GTV Dmax (%)	≤ 130%	114.6	114.9	115.9	116.5	115.8 ± 0.8	114	117.4	129.1	120.2 ± 7.9
PTV D95% (%)	≥ 100%	99.7	95.1	96.3	91.5	94.3 ± 2.5	100.2	100.4	4.5	68.4 ± 55.3
PTV D99% (%)	≥ 95%	90	83.4	85.9	77.3	82.2 ± 4.4	95.1	94.9	3.3	64.4 ± 52.9
PTVOpt V100% (%)	≥ 95%	95.1	88.4	90.9	89.3	89.5 ± 1.3	95.6	95.9	35.7	75.7 ± 34.7
PTVOpt V95% (%)	≥ 95%	98.6	95.2	96	93.2	94.8 ± 1.4	99.2	99.2	53.2	83.9 ± 26.6
GTVOpt V100% (%)	≥ 95%	100	99.6	99.9	96.6	98.7 ± 1.8	100	100	57	85.7 ± 24.8
PTVOpt-GTVOpt Dmax (%)	≤ 110%	110.4	113.6	115.4	115	114.7 ± 0.9	111.8	112.3	129.1	117.7 ± 9.8
Colon V28.8 (cc)	≤ 20 cc	13.84	10.28	14.67	3.91	9.6 ± 5.4	13.04	14.04	0.94	9.3 ± 7.3
Colon D0.04 cc (Gy)	≤ 44 Gy	12.98	12.81	13.26	13.2	13.1 ± 0.2	12.89	13	12.46	12.8 ± 0.3
Heart/Pericardium V24 (cc)	≤ 15 cc	4.3	7.06	13.06	0.08	6.7 ± 6.5	3.76	6.49	6.72	5.7 ± 1.6
Heart/Pericardium D.03 cc (Gy)	≤ 29 Gy	9.55	11.14	11.14	8.21	10.2 ± 1.7	9.49	9.6	9.65	9.6 ± 0.1
Spinal Cord V15.9 (cc)	≤ 0.35 cc	0.01	0	0.6	0.1	0.2 ± 0.3	0	0	0.44	0.2 ± 0.3
Spinal Cord Dmax (Gy)	≤ 22 Gy	5.67	5.56	6.82	5.65	6 ± 0.7	5.26	5.4	6.4	5.7 ± 0.6
Stomach V22.5 (cc)	≤ 5 cc	2.61	6.07	3.63	3.95	4.6 ± 1.3	2.49	2.49	0.15	1.7 ± 1.4
Stomach D0.04 cc (Gy)	≤ 29 Gy	9.15	11.08	10.36	9.98	10.5 ± 0.6	9.44	9.57	7.8	8.9 ± 1
Esophagus V27.9 (cc)	≤ 5 cc	0	0	0	0	0 ± 0	0	0	0	0 ± 0
Esophagus D0.04 cc (Gy)	≤ 32.4 Gy	7.99	8.16	8.73	8.2	8.4 ± 0.3	7.5	8.67	6.92	7.7 ± 0.9
Great Vessels V39 (cc)	≤ 10 cc	0	0	0.01	0	0 ± 0	0	0	0	0 ± 0
Great Vessels D0.04 cc (Gy)	≤ 45 Gy	12.61	12.75	12.87	12.21	12.6 ± 0.4	12.4	12.55	10.86	11.9 ± 0.9
Total Lung V11.4 (%)	≤ 35%	16	17.5	16.5	15.2	16.4 ± 1.2	17.7	21	5.5	14.7 ± 8.2
Chest Wall/Ribs V40 (cc)	≤ 5 cc	0	0	0	0.81	0.3 ± 0.5	0	0.01	0	0 ± 0
Chest Wall/Ribs D0.04 cc (Gy)	≤ 50 Gy	13.1	13.1	13.11	13.52	13.2 ± 0.2	13.01	13.19	13	13.1 ± 0.1

**Figure 2:**
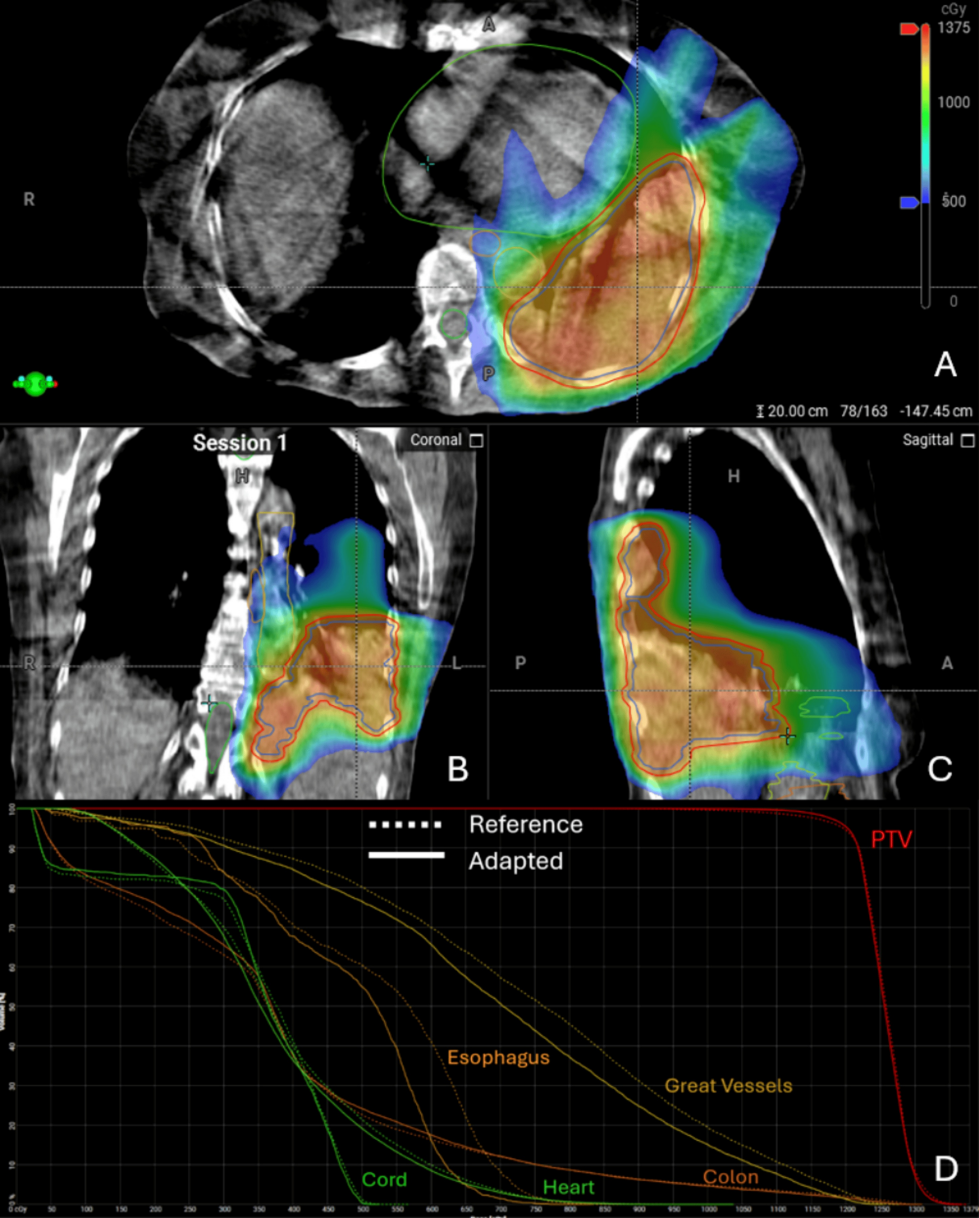
First Fraction of Online Adaptive Radiotherapy to a Bulky Thoracic and Abdominal Mass Cone-beam computed tomography (CBCT) and dose-volume histogram (DVH) from the first fraction of adaptive stereotactic body radiotherapy (SBRT). Dose colorwash with relevant target volumes and avoidance structures contoured on Ethos platform (Varian Medical Systems, Inc., Palo Alto, CA, USA) on axial (A), coronal (B), and sagittal (C) views. DVH (D) for both the selected adapted plan (solid line) and reference plan (dashed line) for planning target volume (PTV; red), great vessels (gold), colon (orange), esophagus (brown), heart (light green), and spinal cord (dark green).

The adapted plan implemented in the initial fraction exceeded all goals of coverage for both GTV and PTV compared to the scheduled plan and the reference plan while overall limiting hotspots, as illustrated by the maximum dose (Dmax) of 114.9% in the scheduled plan and 114.0% in the chosen adapted plan. Moreover, the adapted plan was chosen given its relative decrease in the dose to nearby critical OARs, namely heart, stomach, colon, esophagus, and spinal cord (Figure 2).

**Figure 3:**
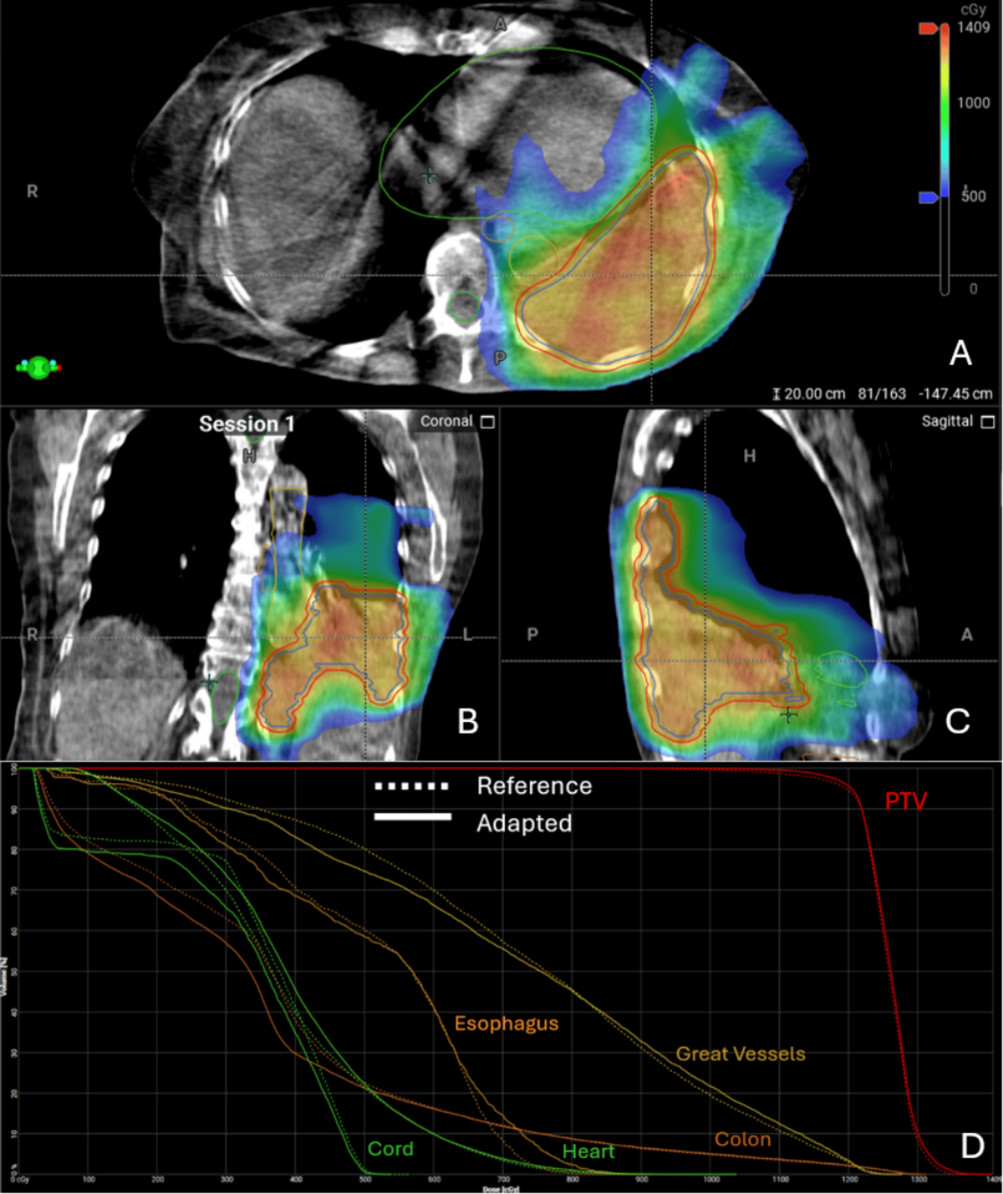
Second Fraction of Online Adaptive Radiotherapy to a Bulky Thoracic and Abdominal Mass Cone-beam computed tomography (CBCT) and dose-volume histogram (DVH) from the second fraction of adaptive stereotactic body radiotherapy (SBRT). Dose colorwash with relevant target volumes and avoidance structures contoured on Ethos platform (Varian Medical Systems, Inc., Palo Alto, CA, USA) on axial (A), coronal (B), and sagittal (C) views. DVH (D) for both the selected adapted plan (solid line) and reference plan (dashed line) for planning target volume (PTV; red), great vessels (gold), colon (orange), esophagus (brown), heart (light green), and spinal cord (dark green).

During the second fraction, a clinically significant change in the tumor volume and shape, as well as OARs, necessitated re-contouring (Figure 3). The adapted plan also had improved PTV coverage, with 100.3% and 94.9% of the PTV receiving at least 95% and 99% of the prescription dose compared to the scheduled plan (96.3% and 85.9%, respectively). Additionally, the adapted plan more effectively limited the dose to OARs than in the scheduled plan.

**Figure 4:**
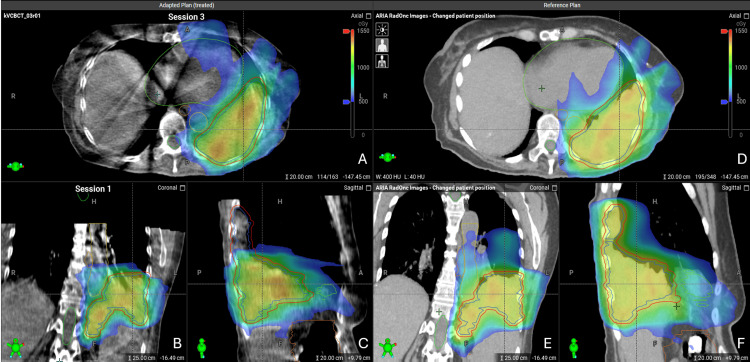
Third Fraction of Online Adaptive Radiotherapy to a Bulky Thoracic and Abdominal Mass Cone-beam computed tomography (CBCT) scan from the third fraction of adaptive stereotactic body radiotherapy (SBRT) compared to reference plan on planning CT (pCT) with dose colorwash overlay. The cumulative point-dose maximum delivered to the heart across the treatments approached the 29 Gray (Gy) maximum dose constraint. Gross tumor volume (GTV) and planning target volume (PTV) coverages were compromised at the discretion of the treating radiation oncologist to protect the heart based on clinical judgment. The axial-adapted CBCT is shown in (A). The superior portion of the mass was blocked to decrease dose to OARs, best seen on coronal (B) and sagittal (C) views. The associated reference plan is shown for comparison with axial (D), coronal (E), and sagittal (F) views. Contours from the adapted plan are displayed on both CBCT and pCT showing GTV (blue), PTV (red), great vessels (gold), colon (orange), esophagus (brown), heart (light green), and spinal cord (dark green).

In the third PULSAR fraction, a CBCT was obtained with the patient on Ethos, and new contours were generated per the oART workflow as described above. However, there were concerns regarding cumulative doses to adjacent OARs given the location and size of the bulky pleural-based mass in the LLL and abdomen. OAR constraints were prioritized in favor of PTV coverage at the discretion of the treating radiation oncologist based on clinical judgment (Figure 4).

**Figure 5:**
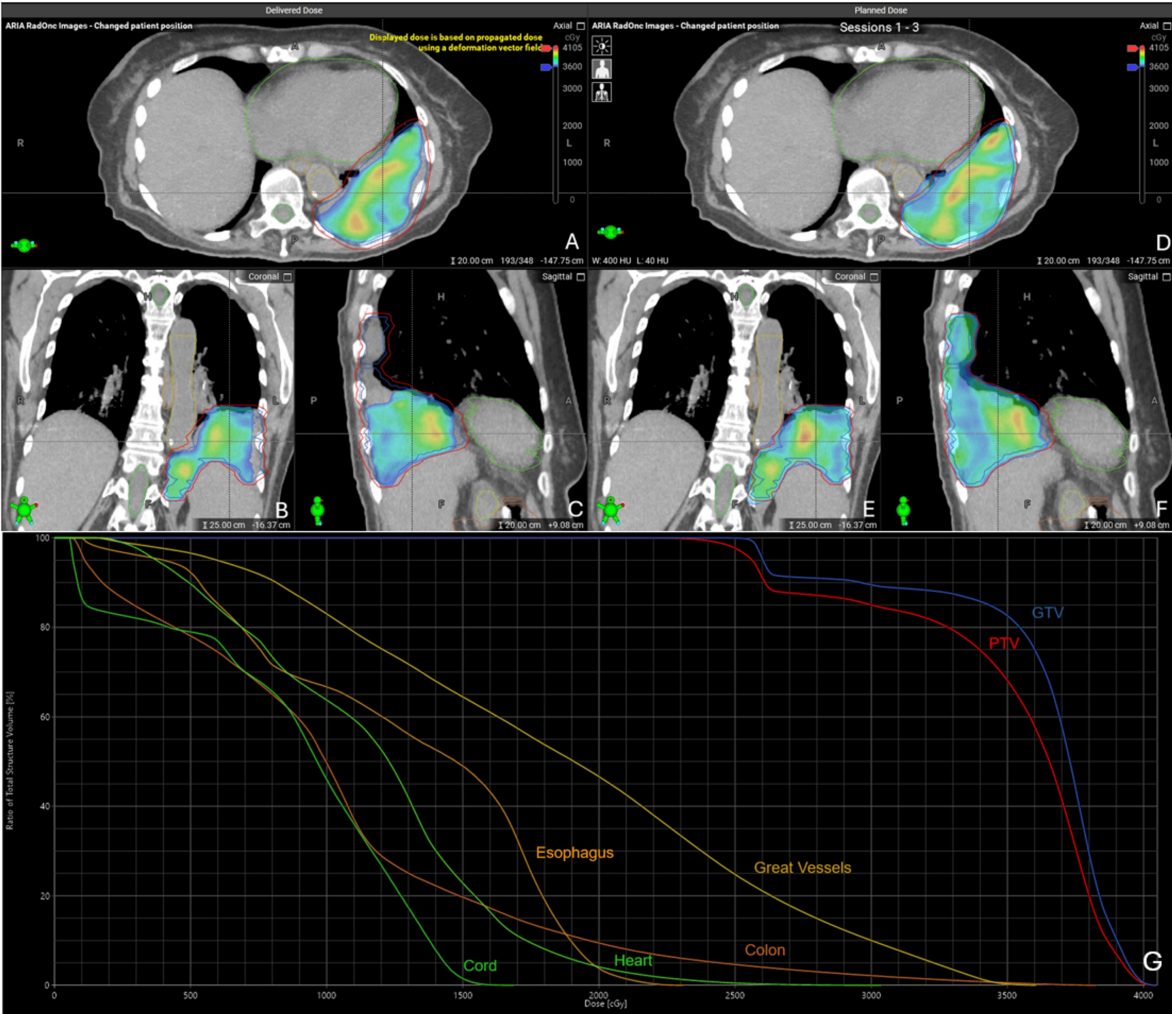
Plan Summary of Online Adaptive Radiotherapy Radiation summary showing delivered and planned dose colorwash overlaid on original planning computed tomography (pCT) scan. Delivered dose is displayed as an accumulation of dose from three fractions displayed on axial (A), coronal (B), and sagittal (C) pCT views. For comparison, the planned total dose is displayed onto the pCT on axial (D), coronal (E), and sagittal (F) views. Cumulative dose-volume histogram (DVH) from three delivered adaptive stereotactic body radiotherapy (SBRT) fractions (G). Contours from original reference plan are displayed with associated DVH curves for gross tumor volume (GTV; blue), planning target volume (PTV; red), great vessels (gold), colon (orange), esophagus (brown), heart (light green), and spinal cord (dark green).

The goal maximum point-dose to the heart was less than 29 Gy, and the cumulative heart point-dose across three PULSAR fractions was 28.74 Gy. Expectantly, there was a dropoff in coverage in the third fraction of PULSAR, and 53.2% and 35.7% of the PTVopt received 95% and 100% of the prescription dose, respectively. In total, 68.4% of the PTV received at least 95% of the prescription dose, and 64.4% received at least 99%. Overall, the adapted plan achieved all point-dose and volumetric constraints to OARs across the three PULSAR fractions. The accumulation of the dose from the adapted plan was compared to the reference plan on the original pCT (Figure 5).

Throughout the course of her adaptive SBRT treatments, there was an observed overall decrease in the size of the target volumes. Between the first and second fractions of monthly PULSAR treatments, there was a 4.6% decrease in the size of the contoured GTV and a 2.7% decrease in PTV (Table 2). Accordingly, there was a decrease in the size of GTVOpt and PTVOpt by 5.1% and 2.8%, respectively. Between the second and third fractions of adaptive SBRT, there was less than 1% change between the contoured target volumes and planning structures.

**Table 2:** Target Structure Volumes Throughout Treatment Absolute value in cubic centimeters (cc) of target structure contours created on planning computed tomography (pCT) scan and cone-beam CT (CBCT) from fraction 1, fraction 2, and fraction 3 of online adaptive radiotherapy.

Structure	pCT (cc)	Fraction 1 (cc)	Fraction 2 (cc)	Fraction 3 (cc)
GTV	322.59	336.44	321.11	321.93
GTVOpt	248.28	259.52	246.32	247.42
PTV	476.93	491.71	478.46	477.84
PTVOpt	474.09	490.76	477.14	477.76

The patient overall tolerated her adaptive SBRT course without developing any grade 3+ radiation-related toxicities. At baseline, she intermittently required supplemental oxygen. After her first two fractions of SBRT, she transiently developed low-grade fever, tachycardia, hypertension, and hypoxia on room air requiring her home level of supplemental oxygen. She additionally had mild nausea and one episode of emesis, which prompted a trip to the local emergency room. Infection work-up was negative, and she was treated with intravenous (IV) fluids, steroids, and anti-emetics. She was discharged with empiric antibiotics and quickly recovered. These symptoms were attributed to a transient systemic response to her overall therapy as she had similar reactions before with systemic therapy alone. She received her third fraction of adaptive SBRT without any adverse events. Based on the Common Terminology Criteria for Adverse Events version 5.0 (CTCAEv5.0), she developed grade 2 nausea and dyspnea due to her adaptive SBRT. The flow of oxygen she required to feel comfortable remained consistent, but the frequency that she needed to use her portable oxygen transiently increased. Additionally, she experienced grade 1 fatigue, cough, anorexia, and weight loss throughout the elapsed three-month period of her PULSAR treatments. These symptoms improved to baseline following completion of her treatment course.

Currently, she is about 10 weeks out from completion of SBRT, and her acute toxicities related to SBRT have resolved. She receives regular follow-up from her multidisciplinary treatment teams, and she has not developed any new or worsening radiation-related toxicities. She underwent CT of her chest, abdomen, and pelvis without IV contrast approximately three weeks following completion of her CT-guided adaptive SBRT to her LLL pleural-based mass extending into the abdomen, and the mass was reported stable in size without any evidence of progression. There were no new areas of metastatic disease.

## Discussion

This case illustrates some of the key advantages and challenges inherent in using online adaptive SBRT when treating a large, bulky, and irregular mass in an area surrounded by dose-limiting OARs and subject to significant changes in tumor size and location. Without oART, SBRT would not have been deemed safe or feasible for a pleural-based mass of this size. For lesions larger than 5 cm, there is limited literature regarding the efficacy of SBRT. Peterson et al. investigated a series of SBRT on non-small cell lung cancer lesions greater than 5 cm in diameter [11]. In general, there were comparable outcomes of local control greater than 90% and distant failure rates slightly above 30% compared to a similar series of SBRT to lung lesions greater than 5 cm [12]. Presumably, most clinicians would be dissuaded from using SBRT on such a large, bulky mass as described in our case report in favor of a more fractionated approach of 30 to 45 Gy in 10 to 15 fractions. Using this approach, the resultant BED would likely be insufficient to provide durable control given the radioresistant nature of RCC. With oART on the Ethos machine and platform, we delivered a BED of 79.2 Gy (α/β=10) for more robust tumor control, and early safety signals are reassuring in this patient’s course.

Furthermore, our patient received monthly fractions of SBRT via PULSAR, and the oART approach allowed for more accurate delineation of target volumes and OARs over the elapsed treatment period. By more closely accounting for changes in shape, size, and proximity to critical structures, a PTV margin of 0.3 cm was used as opposed to the standard 0.5 to 1.0 cm margin that could be applied in a non-adaptive case such as this. Thus, the smaller margins offered by oART potentially minimized the dose to nearby OARs. The adaptive approach also precludes the need for re-simulation, requiring additional planning, staffing hours, and time on the CT scanner.

The adapted plan was selected in favor of the scheduled plan in all three fractions of SBRT delivered to the patient’s pleural-based LLL and upper abdominal mass. In the first two fractions, the adapted plan had superior PTV coverage than the scheduled plan, and it overall limited dose to OARs more effectively. When accounting for OAR constraints to the adjacent critical structures, the PTV dose was decreased in the third fraction to lower the overall heart dose. Although PTV coverage was decreased as a result, the adaptive approach of Ethos allowed greater flexibility in plan delineation, which in turn delivered a robust consolidative dose to the patient’s bulky mass while preserving the dose to nearby critical structures. 

With regard to oncologic outcomes, the patient underwent short-term follow-up diagnostic imaging about three weeks following completion of SBRT, and the radiology report indicated there were stable findings and no evidence of local progression of her pleural-based LLL mass extending into the upper abdomen. She also did not experience any documented grade 3 or higher toxicities related to her oART. However, following her first two fractions of SBRT, she experienced low-grade fever, tachycardia, hypertension, and mild exacerbation of her chronic dyspnea. These adverse reactions were attributed to systemic effects that were also seen with her previous oncologic therapies.

## Conclusions

Although early in the patient's follow-up, the CT-guided adaptive approach generated a safe and effective SBRT plan that was delivered on Ethos, and selection of the adaptive plan mitigated dose overlap with critical structures in the chest and upper abdomen. The oART plan not only delivered the higher BED necessary in oligometastatic RCC, but it also prioritized OAR constraints and decreased the PTV margin compared to a non-adaptive treatment plan. Thus, an adaptive approach was critical for our dose delivery of a metastatic RCC tumor of this size spanning between the thoracic and abdominal cavities.
